# Malignant glomus tumor of the breast: a case report

**DOI:** 10.3389/fonc.2024.1393430

**Published:** 2024-05-09

**Authors:** Qian Mou, Zhenpeng Jiang, Jiaojiao Zhou

**Affiliations:** ^1^ Department of Ultrasound Medicine, West China Hospital of Sichuan University, Chengdu, Sichuan, China; ^2^ Department of Nuclear Medicine, Guangzhou First People’s Hospital, School of Medicine, South China University of Technology, Guangzhou, Guangdong, China

**Keywords:** glomus tumor, malignant, breast, case report, imaging

## Abstract

Malignant glomus tumor (MGT) is a rare mesenchymal neoplasm. It is rarely located in the breast. We present a case of a 57-year-old female patient presenting with complaints of a progressively growing mass found in her left breast. Though multiple imaging examinations have been performed, especially multimodal ultrasound examinations, an accurate diagnosis still cannot be determined. Finally, the lesion was confirmed to be a MGT of the breast by postoperative pathological diagnosis. In conclusion, MGT originating from breast is extremely rare. No such case has ever been described before. This study demonstrates the imaging characteristics of a patient with MGT of the breast in order to provide more extensive insights to consider the differential diagnosis of breast lesions.

## 1 Introduction

Glomus tumors (GTs) are rare mesenchymal neoplasms, accounting for about 2% of soft tissue tumors, and occurring most frequently in the subungual region of the distal extremities (1). Due to its extremely low incidence and lack of characteristic typical imaging features, the diagnosis of GTs mainly relies on histopathology and immunohistochemistry (2). Most GTs are commonly regarded as benign tumors, while malignant glomus tumors (MGTs) are extremely rare, constituting less than 1% of GTs (3). So far, only five cases of GTs occurring in the breast are reported, and all of them are benign. To our knowledge, this is the first case of MGTs originating from the breast.

## 2 Case report

**Figure 1:**
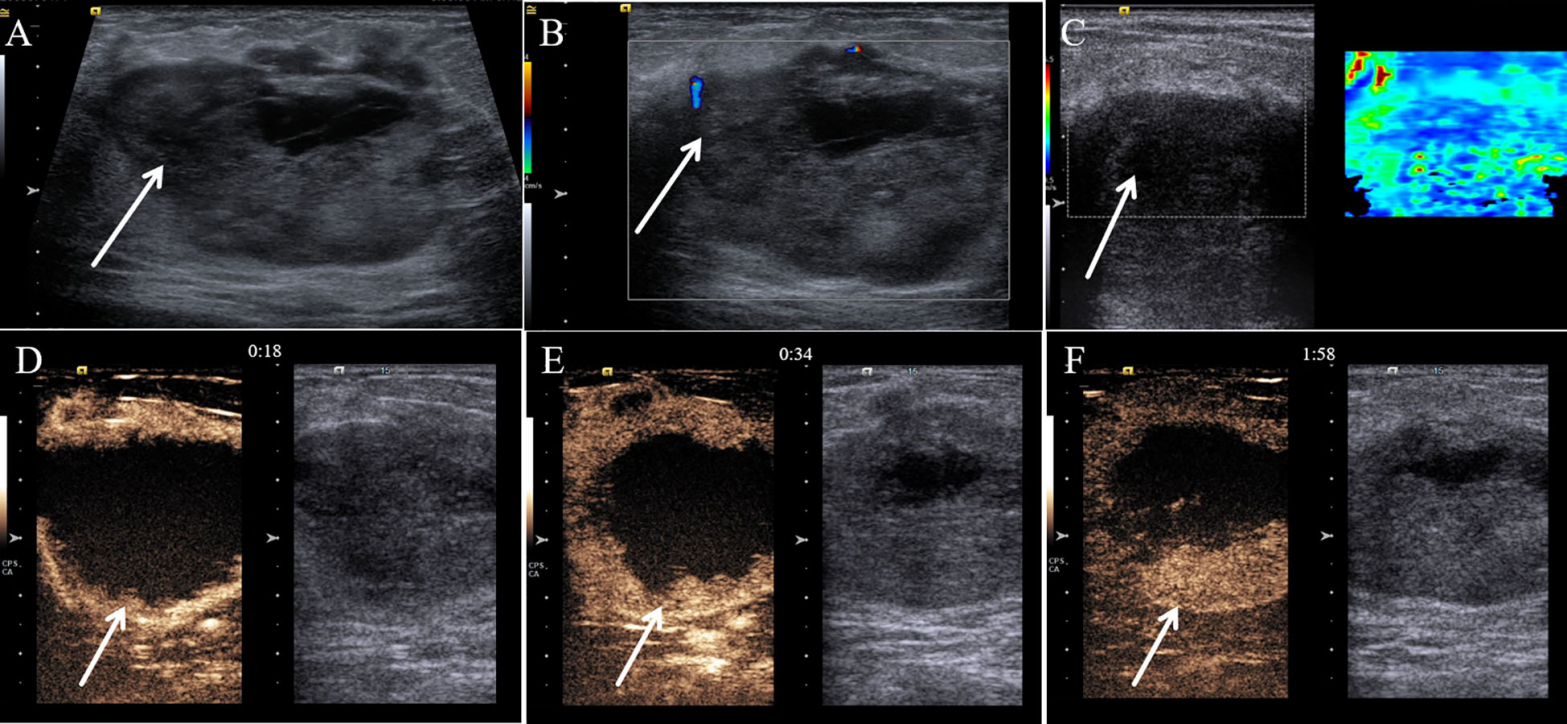
Multimodal ultrasound performance of malignant glomus tumor in the breast. **(A)** Conventional gray-scale sonography revealed a mixed solid and cystic lump (arrow) in the breast. **(B)** Color Doppler flow imaging showed several blood flow signals that were observed in the periphery of the tumor (arrow). **(C)** Shear wave elastography showed a soft nodule (arrow) of the breast. **(D)** The contrast-enhanced ultrasound image captured 18 s after the injection of the contrast agent. **(E)** The contrast-enhanced ultrasound image captured 34 s after the injection of the contrast agent. **(F)** The contrast-enhanced ultrasound image captured 118 s after the injection of the contrast agent.

**Figure 2:**
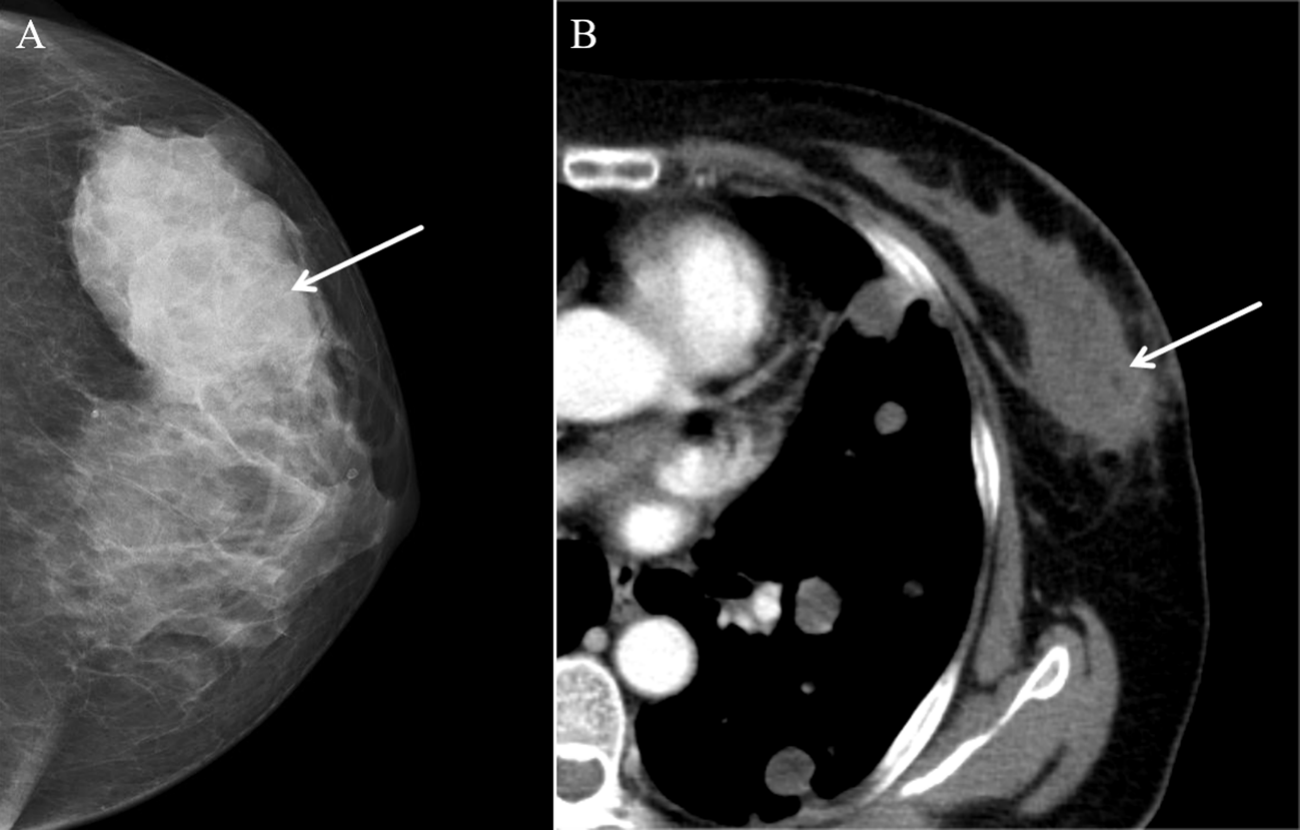
Mammography and contrast-enhanced CT (CE-CT) findings of malignant glomus tumor in the breast. **(A)** Axial mammography image showing a high-density lesion (arrow). **(B)** CE-CT revealed a poorly defined soft tissue mass (arrow).

A 57-year-old female patient presented to our hospital with a 6-month history of a progressively growing left breast mass. The patient had no significant symptoms except for mild tenderness upon the mass. The patient has no family history of breast cancer. Consequently, she has not sought medical attention for the lump promptly. Physical examination revealed a fixed, lobulated, moderately hard mass of approximately 70 mm in diameter in the left breast, with no nipple discharge and nipple retraction. No enlarged axillary lymph node was palpable. Meanwhile, tumor markers such as carcinoembryonic antigen (CEA) and carbohydrate antigen 19-9 (CA 19-9) were within the normal ranges. A mixed solid and cystic lump (measuring 64 mm × 37 mm × 49 mm) with an unclear boundary was found in the external upper quadrant of the left breast, and punctate echogenic foci was not detected on conventional ultrasonography (**Figure 1A**). According to the American College of Radiology, Breast Imaging Reporting and Data System (ACR BI-RADS), this nodule was assigned to BR-4b category. Color Doppler blood flow imaging shows no significant blood flow signal within the nodule, with several blood flow signals observed in the periphery (**Figure 1B**). Spectral Doppler examination showed the resistance index to be 0.74. Moreover, the point shear wave elastography (SWE) assessment of the lesion revealed that the lesion is of low stiffness (**Figure 1C**). Furthermore, contrast-enhanced ultrasound (CE-US) was performed with the injection of SonoVue (Bracco, Milan, Italy). The lesion showed homogeneous hyperenhancement, and its enhancement pattern is centripetal, filling from the periphery toward the center (**Figures 1D–F**). The mammography results indicate that the left breast exhibited heterogeneous density. A high-density mass (**Figure 2A**) measuring approximately 6.5 × 4.5 cm was identified in the upper outer quadrant of the left breast, displaying irregular morphology with clear margins. No abnormal calcifications were observed within the lesion. According to the Breast Imaging Reporting and Data System (BI-RADS), this lesion was classified as BI-RADS 4b. Remarkably, no abnormal axillary lymph nodes were found not only in ultrasound and X-ray examination but also in lymph node scintigraphy. The artificial intelligence analysis of the left breast mammography indicates a high risk of malignancy in the lesion detected in the left breast. In addition, contrast-enhanced computed tomography (CE-CT) not only revealed a poorly defined soft tissue mass (**Figure 2B**) on the outer side of the left breast but also identified numerous lesions (the maximum diameter was 30 mm) in multiple organs, including the lungs, liver, left adrenal gland, both kidneys, pancreas, spleen, and colon. Contrast-enhanced magnetic resonance imaging (CE-MRI) reveals multiple abnormal enhanced lesions (the maximum diameter was 13 mm) in the intracranial and head soft tissues. The radiologist strongly suspects that the aforementioned enhanced lesions outside the breast are metastatic lesions.

**Figure 3:**
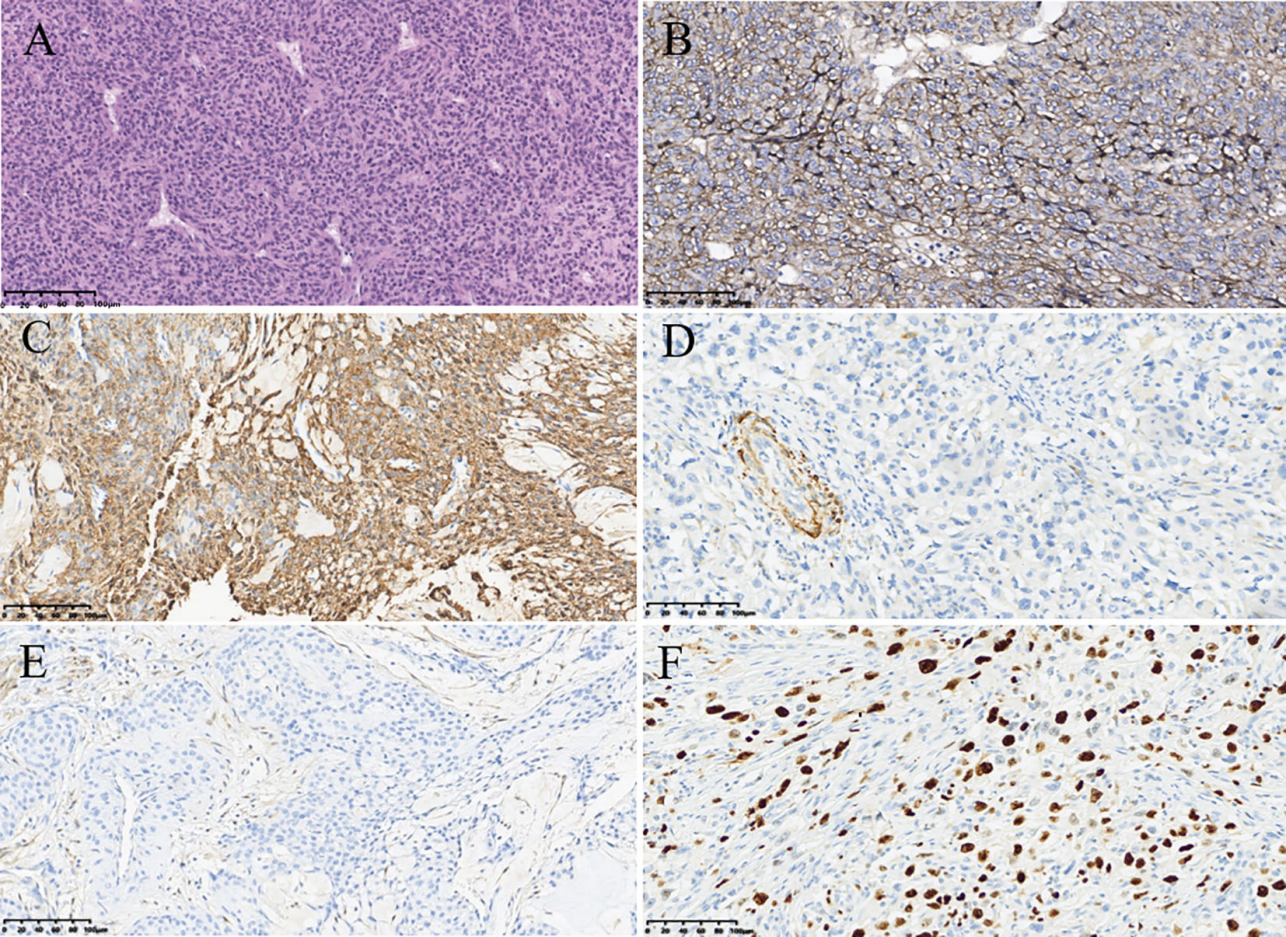
Pathological findings of malignant glomus tumor in the breast. **(A)** Hematoxylin–eosin (magnification, ×200). **(B)** Immunohistochemistry result showing the expression of Collage-IV (magnification, ×200). **(C)** Immunohistochemistry result showing the expression of smooth muscle actin (magnification, ×200). **(D)** Immunohistochemistry result showing the expression of desmin (magnification, ×200). **(E)** Immunohistochemistry result showing the expression of signal transducer and activator of transcription 6 (magnification, ×200). **(F)** Immunohistochemistry result showing the expression of Ki-67 (magnification, ×200).

Ultimately, the patient underwent resection of the lesion of the left breast for further diagnosis. Axillary lymph node dissection was not performed due to the fact that no abnormal lymph node was found by different imaging modalities. This surgery did not remove the lesions outside of the breast. The histologic examination of the tumor showed that it consisted of abundant, regular, oval tumor cells with clear boundaries (**Figure 3A**). Prominently pleomorphic nuclei were observed in the cells of the tumor. On immunohistochemistry, the tumor had strong Collage-IV (**Figure 3B**) and strong smooth muscle actin expression (**Figure 3C**), while desmin (**Figure 3D**), STAT6 (**Figure 3E**), epithelial membrane antigen, S-100 protein, CD31, and CD34 were negative. The expression of Ki-67 (**Figure 3F**) was 60%. Finally, the lesion was confirmed to be a MGT of the breast by histopathology.

After surgery, due to the patient’s refusal of radiation and chemotherapy, targeted therapy with anlotinib was performed. Unfortunately, the patient passed away after 3 months, possibly due to multiple organ failure caused by MGT metastases.

## 3 Discussion

MGT is a rare malignant tumor which has a much lower incidence than its benign counterpart. The majority of MGT mainly occur in the subungual region of the distal extremities, while some MGT can also occur in extracutaneous areas, such as the gastrointestinal tract, lungs, kidneys, and thyroid. To the best of our knowledge, only five benign glomus tumors occurring in the breast were reported in the previous literatures, while we report the first case of MGT arising from the breast (4–8).

MGT has a very high tendency for distant metastases, and the most common sites of metastases are the brain, liver, lung, and lymph nodes (9, 10). In our case, a large number of abnormal lesions are also found in extra-mammary sites, such as the brain, liver, lung, and other organs. Regrettably, we cannot obtain pathological findings due to the patient’s refusal for surgery and puncture operations of extra-mammary lesions. However, metastases were still considered to be the most likely diagnosis according to the imaging findings of those lesions on CE-CT and CE-MRI. Most of the patients with malignant glomus tumor died soon after the diagnosis because of tumor progression and distant metastases (2). The patient in our case died 3 months after diagnosis as well. Therefore, we also speculate that the extra-mammary lesions are metastases originating from MGT of breast. Notably, no abnormal lymph nodes were found in ultrasound, CT, and lymph node scintigraphy. That is why lymph node dissection was not done when the lesion of the left breast was removed. We hypothesized that the principal type of tumor metastasis in our case was hematogenous metastasis instead of lymphatic metastasis. The absence of abnormal lymph nodes also makes it difficult to judge as to whether the tumor is benign or malignant when imaging was performed.

The initial preoperative radiographic diagnosis of MGT can be difficult and error-prone. MGT usually manifests as hypoechoic solid, cystic-solid tumor in conventional ultrasound (11). Those features are consistent with our case. Previous studies regarded that MGTs usually show an abundant blood signal on color Doppler and have a certain diagnostic value (12). However, our case showed a low blood signal. This reminds that the features of MGT on color Doppler may be variable. Our study also describes imaging findings on CE-US and elastography, which are almost absent in the previous literature. MGT, in our case, showed homogeneous hyperenhancement, and its enhancement pattern is centripetal, filling from the periphery toward the center, during the procedure of CE-US. The CE-US findings of MGT are similar to those of some cavernous hemangiomas to some extent. Previous studies have concluded that MGT and hemangioma have comparable imaging characteristics on conventional ultrasound and MRI (13). Therefore, we speculate that the overlap between the imaging manifestations on CE-US of the two is reasonable, and the feature on CE-US may have potential diagnostic significance of MGT. The MGT of our case was soft according to ultrasound elastography. This feature makes the lesion more likely to be misdiagnosed as a benign lesion of the breast. However, there are few available literatures that describe the characteristic imaging features of extradigital MGT, and even fewer reports provide a comprehensive analysis of ultrasonographic features in detail (8). Therefore, further cases are needed to confirm our findings.

Mammography is indeed one of the most commonly used imaging methods to detect breast masses owing to its convenience, affordability, and high sensitivity to calcifications, making it highly favored by clinicians (14). Nonetheless, due to its relatively low sensitivity and the associated risk of ionizing radiation, mammography is primarily utilized for screening purposes (15). Both mammography and traditional ultrasound primarily concentrate on morphological alterations in breast masses, which can potentially lead to misdiagnosis and overlooked cases (16). Tumor angiogenesis is intricately linked with tumor progression, infiltration, and metastasis. By honing in on the distinctive features of tumor microvasculature, the accuracy of disease detection can be significantly enhanced (17). Dynamic contrast-enhanced magnetic resonance imaging (DCE-MRI) stands out as the predominant imaging modality in this domain (18). The extent of enhancement and kinetic parameters derived from DCE-MRI have been shown to correlate closely with the histopathological changes associated with angiogenesis. DCE-MRI exhibits notable sensitivity, surpassing that of mammography and ultrasound imaging, particularly in detecting invasive cancer, with sensitivity levels nearing 100% (17). Moreover, DCE-MRI remains unaffected by factors such as breast tissue density, scar tissue, prior radiotherapy, or breast implants (19). Apart from its diagnostic utility in identifying breast masses, DCE-MRI also serves as a valuable tool for the early prognosis and evaluation of neoadjuvant chemotherapy response in breast cancer cases, thanks to its ability to assess tumor microvascular perfusion (20). Presently, the comprehensive evaluation of tumor vascularity via DCE-MRI has emerged as a pivotal aspect of diagnosing and managing malignant breast tumors. Unfortunately, in the present case, DCE-MRI was not conducted, thereby precluding the assessment of MGT presentation on DCE-MRI. In addition to DCE-MRI, superverb microvascular imaging (SMI) has emerged as a notable technique to evaluate the microvascular supply in breast tumors in recent years. SMI, utilizing innovative Doppler technology, employs multidimensional filtering to segregate blood flow signals from clutter, thus eliminating unwanted artifacts while preserving slow intravascular signals, all without the need for contrast agents (21). Studies have demonstrated that SMI offers superior resolution in depicting microvascular blood flow patterns and the angiogenesis characteristic of malignant breast tumors compared to conventional color Doppler flow imaging and power Doppler imaging (22). Some research findings suggest that SMI can highlight more penetrating vessels in breast cancer cases, aiding in distinguishing between benign and malignant lesions in the breasts without detectable abnormalities, especially those categorized as BI-RADS category 4 breast lesions (21, 23). Nevertheless, further investigations are warranted to ascertain whether the diagnostic efficacy of SMI is comparable to that of CEUS.

MGT is an exceedingly rare stromal tumor. Histologically, MGT typically originates from glomus body cells (3). Glomus bodies, normally found in the dermis and subcutaneous tissue, are contractile neuromyoarterial receptors that regulate blood flow, primarily located in areas such as the palms, wrists, forearms, and beneath toenails (8). However, reports of glomus tumors occurring in atypical locations, such as bone, respiratory tract, cheeks, earlobes, tongue, stomach, sacrum, and buttocks, exist (8). The underlying mechanism remains ambiguous, contributing to the frequent misdiagnosis of MGT in such locations (10). MGT typically comprises numerous round tumors with enlarged nuclei, prominent nuclear division, and tumor cells surrounding blood vessels as they grow (4). Although breast stromal tumors are relatively rare, they encompass a diverse range of entities. From a pathological standpoint, a differential diagnosis of MGT includes glomus tumor, cellular or cavernous hemangioma, and paraganglioma. Glomus tumors are benign, with minimal tumor cell atypia and rare mitotic figures. While focal areas resembling cavernous hemangiomas may be observed in some malignant glomus tumors, hemangiomas typically express thrombomodulin, CD31, and CD34, with negative SMA, aiding in differential diagnosis. Paraganglioma and malignant glomus tumor share histological similarities, but paragangliomas specifically express neuroendocrine markers such as chromogranin A and synaptophysin while lacking SMA expression. Additionally, unlike MGT, solitary fibrous tumors express STAT6, melanomas express S100, and neuroblastomas lack SMA expression (4, 24).

A differential diagnosis of imaging for MGT in our case may mainly include breast carcinoma, breast phyllodes tumor, and breast hemangioma. Breast cancer is the most common malignancy among women (25). It usually presents as a painless, firm, fast-growing mass. As for ultrasonic features, breast cancer usually shows an irregular morphology and indistinct borders with microcalcification and a high aspect ratio in conventional ultrasound, nonhomogeneous enhancement in CEUS, and a stiff mass in SWE (26). These findings were different from our case. Phyllodes tumors of the breast are common breast fibroepithelial neoplasms including benign phyllodes tumor, borderline phyllodes tumor, or a malignant phyllodes tumor (27). The main sonographic appearance of phyllodes tumor is lobulated mass. No significant difference was observed in lesion boundary, orientation, posterior acoustic features, or echo pattern between benign and borderline or malignant phyllodes at sonography (28). The shape of our case is similar to phyllodes tumor to a certain extent, so the lesion and phyllodes tumor cannot be distinguished by sonographic appearances. Hemangioma is a rare benign vascular tumor of the breast (29). It typically presents as a hypoechoic, well-circumscribed oval mass and is located more superficially in the papillary dermis or epidermis (13). Color Doppler usually reveal a rich blood flow signal in hemangioma (4). These findings were different from our case.

## 4 Conclusion

We report an extremely rare case of MGT originating from breast which has never been described before. Due to the low incidence and deep location, ultrasonic manifestations of MGT are rarely reported. Although pathologic confirmation is required for the final diagnosis of MGT, we proposed the performance of MGT in multiple ultrasound modalities, hoping such to be useful in the diagnosis of MGT.

## Data availability statement

The original contributions presented in the study are included in the article/supplementary material. Further inquiries can be directed to the corresponding author.

## Ethics statement

The studies involving humans were approved by Biomedical Ethics Review Committee of West China Hospital, Sichuan University. The studies were conducted in accordance with the local legislation and institutional requirements. The participants provided their written informed consent to participate in this study. Written informed consent was obtained from the individual(s) for the publication of any potentially identifiable images or data included in this article.

## Author contributions

QM: Writing – original draft. ZJ: Writing – original draft. JZ: Writing – review & editing.
